# Residual or Recurrent Precancerous Lesions After Treatment of Cervical Lesions in Human Immunodeficiency Virus–infected Women: A Systematic Review and Meta-analysis of Treatment Failure

**DOI:** 10.1093/cid/ciy1123

**Published:** 2019-01-02

**Authors:** Pierre Debeaudrap, Joelle Sobngwi, Pierre-Marie Tebeu, Gary M Clifford

**Affiliations:** 1 Centre Population et Développement, Institut de Recherche pour le Développement, Université Paris Descartes, INSERM, France; 2 Recherche–Santé & Développement, Cameroon; 3 Department of Gynecology, Centre Hospitalier Universitaire of Yaoundé, Cameroon; 4 Faculty of Medicine and Biomedical Sciences, University of Yaoundé, Cameroon; 5 Interstates School of Public Heath in Central Africa, Congo, Brazzaville; 6 International Agency for Research on Cancer, Lyon, France

**Keywords:** human immunodeficiency virus, human papillomavirus, cervical cancer, treatment failure, meta-analysis

## Abstract

**Background:**

Screening and treating premalignant cervical lesions (cervical intraepithelial neoplasia 2+ [CIN2+]) is an effective way to prevent cervical cancer, and recommendations exist for the monitoring of treatment success. Yet, there is no specific recommendation for human immunodeficiency virus (HIV)-infected women, who are at a known, increased risk of cervical cancer.

**Methods:**

A systematic review was performed by searching MEDLINE, EMBASE, and Web of Science for studies published from January 1980 through May 2018. Eligible studies described the prevalence of histologically- and/or cytologically-defined lesions in HIV-infected women at least 6 months post-treatment. The primary endpoint was treatment failure, defined as the presence of residual and/or recurrent high-grade CIN2+/high-grade squamous intraepithelial lesions post-treatment. The pooled prevalence in HIV-infected women and the odds ratios (ORs) for HIV-infected compared to HIV-uninfected women were estimated using random-effects models.

**Results:**

Among 40 eligible studies, the pooled prevalence of treatment failure in HIV-infected women was 21.4% (95% confidence interval [CI] 15.8–27.0). There was no significant difference in the treatment failure prevalence for cryotherapy (13.9%, 95% CI 6.1–21.6) versus loop electrosurgical excision procedure (13.8%, 95% CI 8.9–18.7; *P* = .9), but the treatment failure prevalence was significantly higher in women with positive (47.2%, 95% CI 22.0–74.0) than with negative (19.4%, 95% CI 11.8–30.2) excision margin (OR 3.4, 95% CI 1.5–7.7). Treatment failure was significantly increased in HIV-infected versus HIV-uninfected women, both overall (OR 2.7, 95% CI 2.0–3.5) and in all sub-group analyses.

**Conclusions:**

There is strong evidence for an increased risk of treatment failure in HIV-infected women, in comparison to their HIV-negative counterparts. The only significant predictor of treatment failure in HIV-infected women was a positive margin status, but further data is needed on long-term outcomes after ablative treatment in HIV-infected women.

## Teaser

Residual or recurrent premalignant cervical lesions are detected in around one-fifth of human immunodeficiency virus (HIV)-infected women treated for precancerous cervical lesions, who are twice as likely to suffer treatment failure as HIV-uninfected women, regardless of the treatment method.

Cervical cancer is the most common cause of cancer and a leading cause of death in women infected with human immunodeficiency virus (HIV) living in limited-resource settings [1, 2]. Women infected with HIV have a higher rate of human papillomavirus (HPV) infection and persistence and an increased risk of cervical precancerous lesions and cancer [3, 4]. The main approaches for treating cervical intra-epithelial neoplasia grade 2–3 (CIN2+) precancerous lesions include excisional (loop electrosurgical excision procedure [LEEP], cold knife conization) and ablative (cryotherapy, laser therapy, thermal ablation) treatments. The former has been extensively used in high-resource countries, because they can provide histological confirmation, while cryotherapy and thermal ablation are favored in limited-resource countries, because of their ease of use and lower costs. The risk of recurrence or persistence after LEEP treatment is around 10%, and is increased in patients with positive margins [5, 6]. After ablative treatment, recurrence rates of similar magnitudes have been reported, but data are more limited. In high-resource settings, the recommendations for follow-up after LEEP are to conduct visits with HPV testing and/or cytology at 6- to 12-month intervals after treatment until negative results are obtained [7]. There is no specific recommendation for HIV-infected women, despite their increased risk of cervical disease, and there is also little evidence for ablative treatment in limited-resource contexts [7]. With the scale-up of cervical cancer screening programs for HIV-infected women, there is an urgent need to better understand the risk of residual/recurrent lesions in those women treated for a precancerous lesion, in order to determine the most appropriate strategy for this population [8]. To address this important issue, we performed a systematic review and meta-analysis of studies reporting the prevalence of cervical lesions after excisional or ablative cervical treatment among HIV-infected women.

## METHODS

### Search Strategy and Selection Criteria

Studies were identified by searching the electronic databases MEDLINE, EMBASE, and Web of Science (Science Citation Index) for studies published between 1 January 1980 and 1 May 2018 that reported rates of cervical lesions after excisional or ablative treatment among HIV-infected women. The search strategy is reported in Supplementary Data S1. The Cochrane Database of Systematic Reviews and the databases of large conferences (International Acquired Immunodeficiency Syndrome Conference, Conference on Retroviruses and Opportunistic Infection, International Conference on AIDS and STIs in Africa, and Conférence Internationale Francophone VIH, Hépatites et Santé Sexuelle) were also searched. In addition, the references of retrieved papers were searched for additional publications. Searches were performed without limits to language or setting.

### Article Selection and Data Extraction

Independently, 2 authors (P. D. and J. S.) assessed the eligibility of each paper. Disagreements were resolved by consensus. To be included, a study had to meet the following, prespecified criteria: (1) describe the treatment of cervical abnormalities, (2) report the prevalence of cervical neoplastic lesions, identified through histological and/or cytological methods, at least 6 months post-treatment, and (3) include the above measures for HIV-infected women. Any treatment modality among excisional and ablative methods was eligible. Studies were not included if they reported exclusively on the treatment of invasive cervical cancer or used only visual inspection with acetic acid (VIA) to detect post-treatment lesions. Data extraction was done independently by 2 reviewers, using a standardized data extraction form and recording: (1) study characteristics, (2) participant characteristics, (3) cervical lesion severities at treatment, (4) treatment modalities, (5) outcome details (including methods of detection and severities of outcomes), and (6) in studies reporting on outcomes after excisional treatment, the histological status of the excision margin. Results were extracted for the post-treatment presence of high-grade lesions (CIN2+/high-grade squamous intraepithelial lesions [HSIL+]) and of cervical lesions of any grade (CIN1+/ low-grade squamous intraepithelial lesions [LSIL+]), and both results were recorded when available.

### Outcome Variables

The primary outcomes considered in this analysis were the prevalence of treatment failure (defined as residual and/or recurrent CIN2+/HSIL+ lesions post-treatment [5]) in HIV-infected women and the odds ratio (OR) for treatment failure in HIV-infected compared to HIV-uninfected women.

Secondary outcomes included the presence of any grade of cervical lesion post-treatment (including CIN1+/LSIL+) in HIV-infected women and the corresponding OR in HIV-infected compared to HIV-uninfected women.

### Statistical Analysis

The proportions of treatment failure (and any grade cervical lesion post-treatment) were computed from the raw data and pooled using a random-effects model to account for the expected heterogeneity across studies. The Clopper-Pearson method was used to compute 95% confidence intervals (CIs). A pooled OR of treatment failure in HIV-infected women compared to HIV-uninfected women was estimated with a random-effects model using the restricted maximum likelihood method [9].

Heterogeneity was assessed with the I^2^ statistic, where an I^2^ value of 50–75% is significant and a value >75% is considerable [10]. The reporting bias was assessed with funnel plots and Egger tests [11].

Sub-group analyses were performed, stratified by treatment modality (LEEP, cryotherapy, mixed/other); type of lesion initially treated (low-grade only: CIN1/LSIL; high-grade only: CIN2+/HSIL+; any grade, including any VIA-detected abnormalities, when a grade distinction was not possible); duration of follow-up (median/mean 6–12 versus >12 months); income setting (high or low); publication year (before versus after 2005, at which time the WHO recommendations were published); whether or not HIV-uninfected women were compared in the same study; and, for a sub-set of studies, LEEP margin status.

## RESULTS

### Study Characteristics

**Figure 1.**
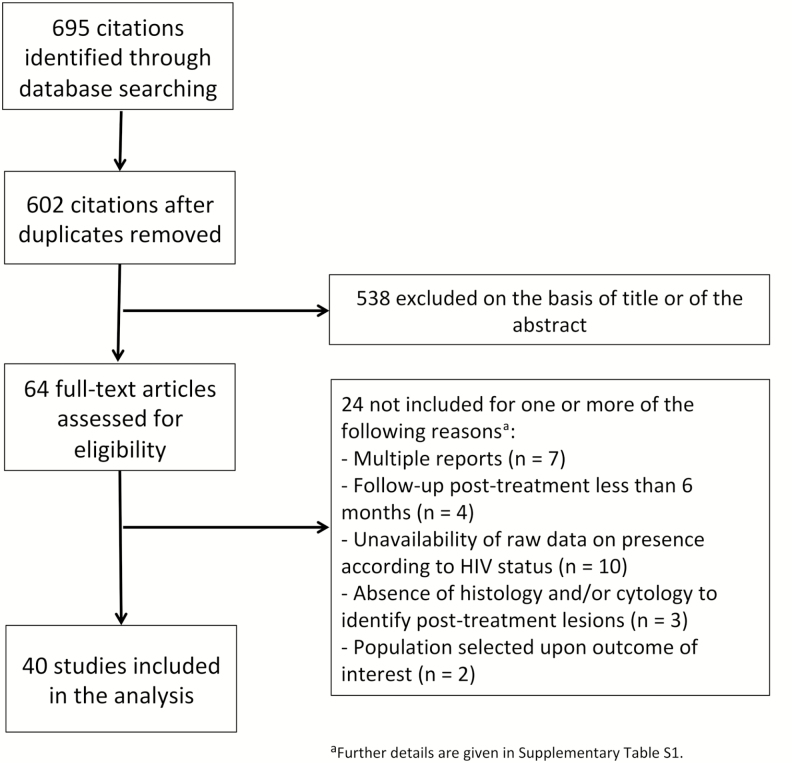
Selection of the studies. Abbreviation: HIV, human immunodeficiency virus.

The electronic search identified 695 publications. After a review of titles and abstracts, a total of 64 full-test articles were assessed for eligibility, of which 24 were excluded for 1 or more of the following reasons (Figure 1 and Supplementary Table S1): multiple reports of the same study (n = 7), follow-up duration <6 months (n = 4), insufficient data on post-treatment outcomes in HIV-infected women (n = 10), no cytological and/or histological ascertainment of post-treatment outcomes (n = 3), or selected study populations (n = 2). A total of 40 studies were eventually included (4 clinical trials, 16 observational cohorts, and 20 retrospective studies), providing data for 3975 HIV-infected women. Of them, 24 reported on high-grade treatment failure (CIN2+/HSIL+) and 34 on any grade (CIN1+/LSIL+) of cervical lesions post-treatment (18 reported on both outcomes). Approximately half of the studies (n = 22) also included HIV-uninfected women (n = 3638) treated in the same setting. The study characteristics are given in Table 1. There was a wide range of follow-up durations across studies, with 27 (69%) having a mean/median follow-up duration >12 months. Patients were treated for high-grade (CIN2+/HSIL+) lesions only in 12 studies (30%), for low-grade (CIN1 and/or LSIL) lesions only in 2 studies, and for cervical lesions of any grade in 26 studies (65%; including 2 studies in which any abnormality detected using VIA were treated). A majority of studies (n = 27) were performed in high-resource settings (mainly the United States). Studies from low- and middle-income countries (n = 13) were often conducted in Southern or Eastern Africa and, compared to those in high-resource countries, were larger (the meta-analysis representing, in the majority, women treated in low-income countries, n = 2460), more often conducted during the last decade (*P* = .03), and more likely to have mean/median follow-up durations of 6–12 months (*P* < .0001). LEEP was the treatment modality most commonly reported by studies from both high- and low-income countries (LEEP was used in 22 and 9 studies from high- and low-income countries, respectively: *P* = .4; cryotherapy in 7 and 6, *P* = .3).

**Table 1.** Characteristics of Included Studies

	Setting and Period	Treatment Procedure	Type of Lesion Treated^a^	Severity of Post-treatment Lesion Detected^a^	Detection Method for Post-treatment Lesions	Mean/Median Follow-up^c^	No. HIV+	No. HIV-
Babkina et al, 2015, J Low Genit Tract Dis ^[21]^	United States (2000–2011)	Mixed/other	High-grade	High-grade	Cyto & histo	>12 mos	44	44
Berrebi, 2008, Gynecol Obstet Fertil [22]	France (<2008)	Mixed/other	High-grade	Any grade	Cyto & histo	>12 mos	22	62
Carlander et al, 2018, AIDS ^[23]^	Sweden (1983–2014)	Mixed/other	High-grade	Any grade; high-grade	Cyto & histo	>12 mos	140	284
Cejtin, 2011, J Low Genit Tract Dis [24]	United States (2004–2009)	LEEP	Any grade	Any grade	Cyto & histo	6–12 mos	70	487
Cejtin, 2017, J Low Genit Tract Dis [25]	United States (2008–2014)	LEEP	Any grade	High-grade	Cyto & histo	>12 mos	34	153
Chirenje, 2003, J Low Genit Tract Dis [26]	Zimbabwe (1997–1998)	Mixed/other	High-grade	Any grade; high-grade	Cyto & histo	6–12 mos	92	32
Cui et al, 2017, Gynecol Oncol ^[27]^	United States (2001–2014)	LEEP	High-grade	High-grade	Cyto & histo	>12 mos	32	417
De Vuyst et al, 2014, PLOS One ^[28]^	Kenya (2009)	Cryo	High-grade	Any grade; high-grade	Cyto & histo	6–12 mos	79	...
Dos Santos Melli, 2016, Int J Gynaecol Obstet [29]	Brazil (2003–2011)	LEEP	High-grade	Any grade; high-grade	Cyto & histo	6–12 mos	85	222
Firnhaber, 2017, JAIDS [30]	South Africa (2012–2015)	Cryo	Low grade	Any grade; high-grade	Cyto & histo	6–12 mos	99	...
Foulot, 2008, Eur J Obstet Gynecol Reprod Biol [31]	France (1993–2006)	Mixed/other	High-grade	Any grade; high-grade	Cyto & histo		61	...
Fruchter, 1996, Obstet Gynecol [32]	United States (1988–1993)	Mixed/other	Any grade	Any grade	Cyto & histo	>12 mos	127	193
Gilles et al, 2005, Gynecol Oncol ^[33]^	Belgium (1995–2002)	Mixed/other	Any grade	Any grade; high-grade	Cyto	>12 mos	57	50
Gingelmaier, 2007, Anticancer Res [34]	Germany (2004–2011)	Mixed/other	Any grade	Any grade	Cyto & histo	>12 mos	70	...
Heard, 1995, Obstet Gynecol [35]	France (1991–1999)	Mixed/other	Any grade	Any grade; high-grade	Cyto & histo	>12 mos	13	...
Heard, 2005, J Acquir Immune Defic Syndr [36]	France (1993–2003)	Mixed/other	Any grade	Any grade High-grade	Cyto & histo	>12 mos	75	...
Holcomb, 1999, Gynecol Oncol [37]	United States (1991–1998)	Mixed/other	Any grade	Any grade	Cyto & histo	>12 mos	66	...
Huchko, 2015, J Acquir Immune Defic Syndr [38]	Kenya (2008–2012)	LEEP	High-grade	High-grade	Histo	6–12 mos	284	...
Kabir, 2012, S Afr Med J [39]	South Africa (2003–2006)	Mixed/other	Any grade	Any grade; high-grade	Cyto	6–12 mos	306	...
Kietpeerakool, 2006, Int J Gynecol Cancer [40]	Thailand (1998–2004)	LEEP	Any grade	Any grade; high-grade	Cyto & histo	6–12 mos	34	...
Kuhn, 2010, AIDS [41]	South Africa (2000–2006)	Cryo	VIA+	Any grade; high-grade	Cyto & histo	>12 mos	105	386
Lehtovirta, 2008, Int J STD AIDS [42]	Finland (1989–2006)	LEEP	Any grade	Any grade	Cyto & histo	>12 mos	34	...
Lima, 2009, Int J Gynaecol Obstet [43]	Brazil (1999–2004)	LEEP	Any grade	Any grade	Cyto & histo	>12 mos	94	107
Lofgren, 2015, AIDS Res Hum Retroviruses [44]	United States (2004–2011)	Mixed/other	Any grade	Any grade; high-grade	Cyto & histo	>12 mos	34	...
Maiman, 1993, Obstet Gynecol [45]	United States (<1993)	Mixed/other	Any grade	Any grade	Cyto & histo	>12 mos	44	125
Maiman, 1999, Obstet Gynecol [46]	United States (1993–1997)	Mixed/other	High grade	Any grade; high-grade	Cyto & histo	>12 mos	51	...
Massad et al, 2007, J Low Genit Tract Dis ^[47]^	United States (1994–2002)	Mixed/other	Any grade	Any grade; high-grade	Cyto & histo	>12 mos	170	...
Moya & Martínez Escoriza, 2012, Clin Invest Gin Obst ^[48]^	Spain (1999–2009)	Mixed/other	Any grade	Any grade	Cyto & histo	>12 mos	9	194
Nappi et al, 2005, Eur J Obstet Gynecol Reprod Biol ^[49]^	Italy (1990–1997)	LEEP	Low-grade	Any grade	Cyto & histo	>12 mos	47	45
Orang’o et al, 2017, AIDS ^[50]^	Kenya (2011–2013)	Cryo	VIA+	High-grade	Cyto, VIA, HPV, & histo^b^	6–12 mos	248	...
Reimers et al, 2010, Gynecol Oncol ^[51]^	United States (1999–2005)	Mixed/Other	Any grade	Any grade; high-grade	Cyto & histo	>12 mos	56	...
Robinson, 1998, Int J Gynecol Cancer [52]	United States (<1998)	LEEP	Any grade	Any grade	Cyto & histo	6–12 mos	8	114
Robinson, 2001, Am J Obstet Gynecol [53]	United States (<2001)	Mixed/other	Any grade	Any grade	Cyto & histo	>12 mos	19	35
Russomano, 2013, Sao Paulo Med J [54]	Brazil (1996–2010)	LEEP	High-grade	High-grade	Cyto & histo	>12 mos	60	209
Shah, 2008, J Obstet Gynaecol [55]	United Kingdom (1995–2004)	Mixed/other	Any grade	Any grade; high-grade	Cyto & histo	6–12 mos	53	...
Smith et al, 2017, Am J Obstet Gynecol ^[14]^	South Africa (2010–2014)	Mixed/other	High-grade	Any grade; high-grade	Cyto, histo, VIA, & HPV DNA^b^	6–12 mos	157	...
Spinillo, 1992, Eur J Obstet Gynecol Reprod Biol [56]	Italy (<1992)	Mixed/other	Any grade	Any grade	Cyto & histo	>12 mos	22	...
Tate, 2002, Am J Obstet Gynecol [57]	United States (1996–2000)	Mixed/other	Any grade	Any grade	Cyto & histo	>12 mos	38	65
Wright, 1994, Gynecol Oncol [58]	United States (1991–1992)	LEEP	Any grade	Any grade	Cyto & histo	6–12 mos	34	80
Zeier, 2012, Int J STD AIDS [59]	South Africa (2004–2009)	Mixed/other	Any grade	Any grade	Cyto & histo	>12 mos	652	309

Mixed/other indicates the use of different treatments, including cryotherapy, thermal ablation, or LEEP and/or other treatments.

Abbreviations: +, positive; -, negative; cryo, cryotherapy; cyto, cytology; histo, histology; HIV, human immunodeficiency virus; HPV, human papillomavirus; LEEP, loop electrosurgical excision procedure; VIA, visual inspection with acetic acid.

^a^A high-grade lesion was defined as a cervical intraepithelial neoplasia 2+ or high-grade squamous intraepithelial lesion.

^b^Any positive VIA, cytology, or HPV test confirmed with histology.

^c^Mean or median follow-up duration of the overall population, if available, or of the HIV-infected participants.

### Treatment Failure in Human Immunodeficiency Virus–infected Women

**Figure 2.**
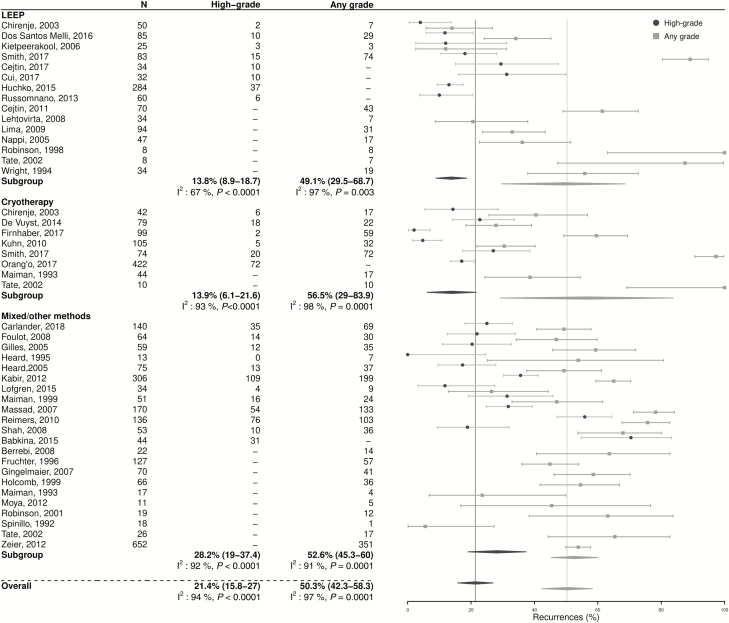
Post-treatment prevalence of cervical lesions in human immunodeficiency virus–infected women, by individual study and treatment modality. Mixed/other indicates the use of different treatments, including cryotherapy, thermal ablation, or LEEP and/or other treatments. Abbreviation: LEEP, loop electrosurgical excision procedure.

The pooled estimate of treatment failure among HIV-infected women was 21.4% (95% CI 15.8–27.0; n = 24; Figure 2), with substantial heterogeneity across studies (I^2^: 94%, *P* < .0001). Treatment failure rates in the studies reporting on cryotherapy use (13.9%, 95% CI 6.1–21.6; n = 6) were not significantly different from those found in studies reporting on LEEP (13.8%, 95% CI 8.9–18.7; n = 8; *P* = .9). The pooled prevalence of any grade of cervical lesion post-treatment among HIV-infected women was higher (50.3%, 95% CI 42.3–58.3; n = 34; I^2^: 96%; *P* < .0001), and was also similar after either cryotherapy (56.5%, 95% CI 29.0–83.9; n = 7) or LEEP (49.1%, 95% CI 29.5–68.7; n = 11; *P* = .7).

**Figure 3.**
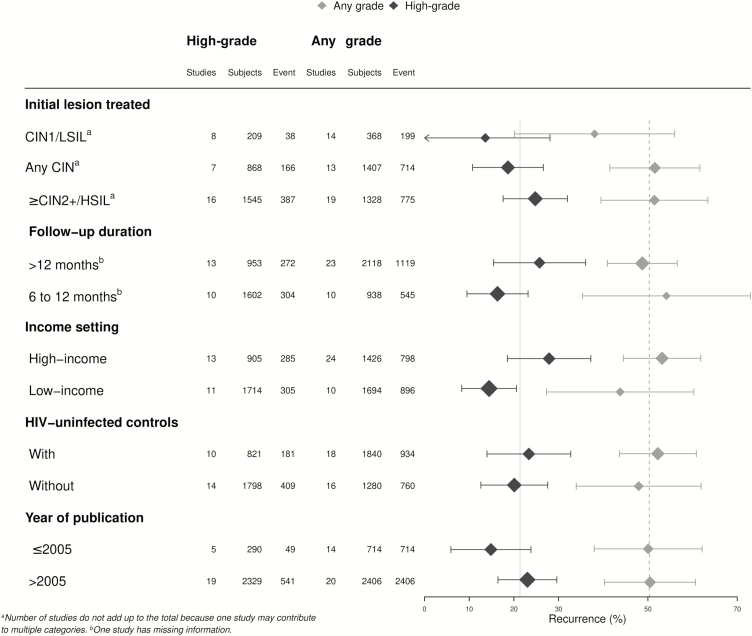
Post-treatment prevalence of cervical lesions in HIV-infected women, by sub-group. Abbreviations: CIN, cervical intraepithelial neoplasia; HIV, human immunodeficiency virus; HSIL, high-grade squamous intraepithelial lesion; LSIL, low-grade squamous intraepithelial lesion.

**Figure 4.**
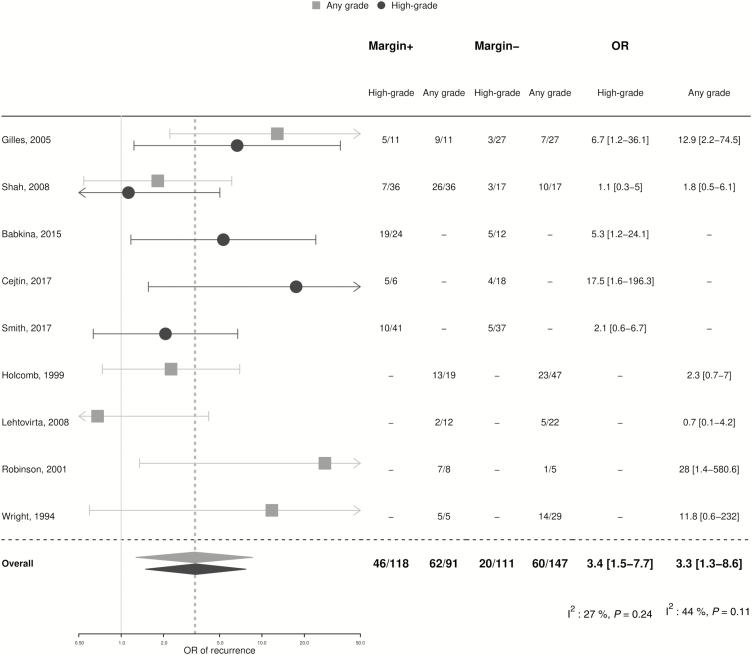
Post-treatment prevalence of cervical lesions in human immunodeficiency virus–infected women, by margin status. Abbreviations: +, positive; -, negative; OR, odds ratio.

Results from sub-group analyses were broadly consistent with the overall estimates (Figure 3). The pooled proportion of treatment failure among HIV-infected women treated for high-grade lesions was 24.8% (95% CI 17.6–32.0; n = 16; I^2^: 92%; *P* < .0001), a proportion that was higher, but not statistically different (*P* = .2), from women treated for CIN1/LSIL (13.6%, 95% CI 0–28.1). The treatment failure proportion in studies with >12 months of follow-up was 25.7% (95% CI 15.4–36.1; n = 13; I^2^: 95%; *P* < .0001), and did not differ significantly (*P* = .1) from studies with shorter follow-up durations (16.3%, 95% CI 9.5–23.2). There was a lower pooled proportion of treatment failure in the studies from low-income (14.4%, 95% CI 8.3–20.6) versus high-income settings (27.9%, 95% CI 18.5–37.2; *P* = .02). Results did not differ between those studies including only HIV-infected participants and those including both HIV-infected and uninfected women (*P* = .6), nor by publication year (*P* = .2). In a sub-group analysis limited to HIV-infected women treated for high-grade lesions only (Supplementary Figure S1), there was a higher risk of treatment failure after cryotherapy (21.6%, 95% CI 14.5–28.6; n = 3) compared to LEEP (12.6%, 95% CI 7.5–17.7; n = 6) that was of borderline statistical significance (*P* = .05). Of note, the highest treatment failure was observed in the sub-group that received mixed/other modalities (31.6%, 95% CI 20.3–42.9). There were 9 studies of excisional treatment that provided information on the presence of post-treatment lesions by margin status. The treatment failure rate was 49.1% (95% CI 22.1–76.1) in HIV-infected women with positive margins and 16.4% (95% CI 9.1–23.7) in those with negative margins, resulting in a pooled OR of 3.4 (95% CI 1.5–7.7; Figure 4). A similar OR of 3.3 (95% CI 1.3–8.6) was found for the presence of any grade of cervical lesion post-treatment.

### Comparison With Human Immunodeficiency Virus–uninfected Women

**Figure 5.**
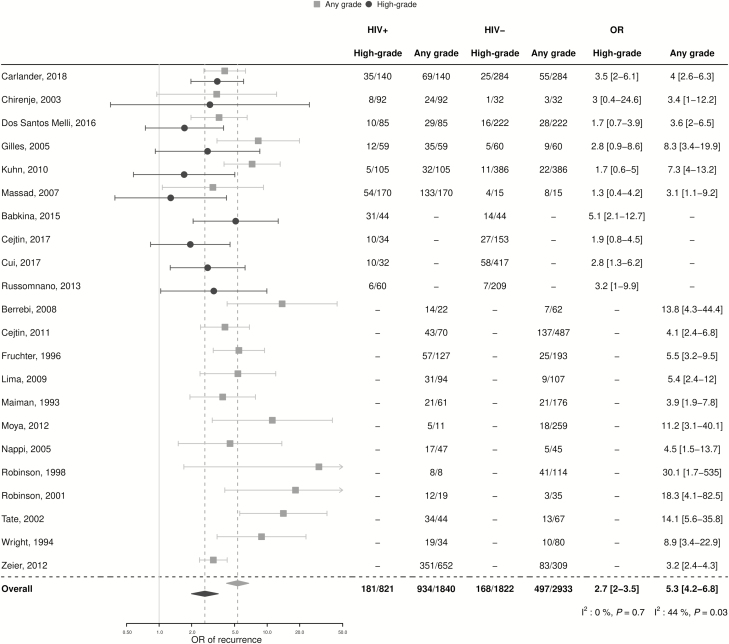
Meta-analysis of the risk of post-treatment lesions after first treatment in HIV-infected versus uninfected women. Abbreviations: +, positive; -, negative; HIV, human immunodeficiency virus; OR, odds ratio.

In the 10 studies with results on treatment failure for both HIV-infected and -uninfected women (n = 821 HIV+ and 1822 HIV-), HIV-infected women had a 2-fold higher risk of treatment failure compared to HIV-infected women (23.4% [95% CI 14.0–32.7] versus 9.5% [95% CI 5.8–13.2], respectively; OR 2.7, 95% CI 2.0–3.5; I^2^: 0%; *P* = .7; Figure 5). Funnel plots did not suggest small-study effects (Supplementary Figure S2, Egger’s test; *P* = .4).

**Figure 6.**
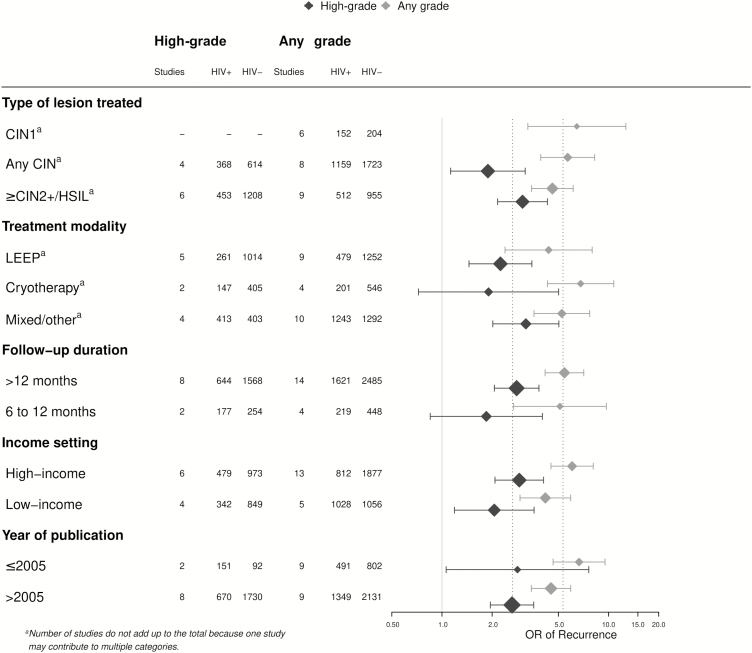
Meta-analysis of the risk of post-treatment lesions after first treatment in HIV-infected versus -uninfected women by sub-group. Mixed/other indicates the use of different treatments, including cryotherapy, thermal ablation, or LEEP and/or other treatments. Abbreviations: +, positive; -, negative; CIN, cervical intraepithelial neoplasia; HIV, human immunodeficiency virus; HSIL, high-grade squamous intraepithelial lesion; LEEP, loop electrosurgical excision procedure; OR, odds ratio.

In sub-group analyses, pooled ORs were highly consistent with overall estimates (Figure 6). For example, when analyses were stratified by treatment modality, the pooled OR of treatment failure was 2.2 (95% CI 1.5–3.5; n = 5; I^2^: 0%; *P* = .9) for LEEP and 1.9 (95% CI 0.7–5.0; n = 2; I^2^: 0%; *P* = .7) for cryotherapy. Pooled ORs were 3.0 (95% CI 2.2–4.3) among women treated for CIN2+/HSIL lesions (n = 6) and 2.8 (95% CI 2.1–3.8) for studies with follow-up durations >12 months (n = 8).

There were 18 studies with results on any grade of cervical lesion post-treatment in both HIV-infected and -uninfected women. The pooled OR of any grade of cervical lesion in HIV-infected versus -uninfected women was 5.3 (95% CI 4.2–6.8; I^2^: 44%; *P* = .3) overall (Figure 5). Sub-group analyses were highly consistent with overall estimates. For example, the estimates were 4.4 (95% CI 2.4–8.0; n = 9; I^2^: 68%; *P* = .002) after LEEP, 6.8 (95% CI 4.3–10.8; n = 4; I^2^: 0%; *P* = .5) after cryotherapy, and 5.4 (95% CI 4.2–7.1; n = 14; I^2^: 50%; *P* = .02) in the subset of studies with mean/median follow-up durations >12 months.

## DISCUSSION

This meta-analysis provides evidence that, even after cervical screening and treatment, women infected with HIV remain at high risk of CIN2+/HSIL cervical lesions. These results complement the knowledge that HIV-infected women have high background risks of HPV infections, precancerous lesions, and cervical cancer [4]. In the context of increasing efforts to scale up cervical cancer screenings in limited-resource settings, these findings highlight the importance of reflecting upon the appropriate post-treatment follow-up of this population.

Our results are in line and extend those of 2 previous reviews: the first focused on limited-resource countries and identified only 4 studies [12] and the second focused only on those studies using excisional methods from high-resource countries, conducted before widespread antiretroviral therapy was available [13]. In limited-resource settings, the use of excisional methods is restricted by the requirements in terms of equipment and training and the inability to perform treatments and screenings in the same visit. This has prompted a shift towards the use of ablative methods. Overall, we did not find evidence for significantly worse outcomes after cryotherapy compared to excisional LEEP in HIV-infected women, which is in agreement with the largest randomized trial on this topic, which found no statistical difference in CIN2+ lesions post-treatment between LEEP and cryotherapy at 12 months [14]. However, there was some suggestion of higher treatment failure rates after cryotherapy among the sub-group of women treated for high-grade lesions. Results of this sub-group analysis should be interpreted with caution, because of the limited number of studies (3 for cryotherapy, 6 for LEEP), but this highlights the continuing need to collect evidence on treatment outcomes after ablative treatment in HIV-infected women [15, 16]. None of the included studies reported on the risk of treatment failure in HIV-infected women after thermal ablation [17]. There were 2 such studies identified by our literature review, but they were ineligible, as they judged treatment failure based on VIA only [15, 18]. Hence, more reporting of the long-term efficacy of this method in HIV-infected women is warranted [17, 19]. Among HIV-infected women treated with LEEP, margin status was associated with the risk of post-treatment lesions, which is in agreement with a recent meta-analysis conducted for predominantly HIV-uninfected women [5]. However, only very few (n = 4) of the included studies reported on positive margin statuses in both HIV-infected and -uninfected women, precluding a meta-analytical comparison at this time.

Limitations of our meta-analysis include important differences regarding the designs, population characteristics, and follow-up procedures of included studies. For instance, 17 of 40 studies did not distinguish the presence of high-grade from low-grade lesions. Although high-grade lesions are more relevant for treatment failure, from a clinical point of view, in the interest of completeness and to avoid selection bias, we did not exclude such studies, but rather chose to present results in parallel for high-grade lesions (as our definition of treatment failure) and cervical lesions of any grade. Furthermore, although most studies used both histology and cytology to confirm the presence of high-grade lesions, outcomes were sometimes based on cytology only.

Other factors that differed by study included the treatment modality (LEEP, cryotherapy, mixed/other), type of lesions treated, and the follow-up durations, which may have contributed to the high heterogeneity observed for the pooled proportion estimates. On the other hand, no significant differences in treatment failure were identified by sub-group analyses according to these variables. Interestingly, the heterogeneity of the risks of treatment failure in HIV-infected compared to HIV-uninfected women was low, and the pooled estimates were not significantly altered in a sub-group analysis, suggesting that the finding of excess treatment failure in HIV-infected women is highly robust. Moreover, our estimate of the proportion of treatment failure in HIV-uninfected women was close to those from larger meta-analyses in this population, suggesting a fair representativeness of included studies [5, 20].

It was not possible to stratify analyses by CD4-cell count (CD4), because of the lack of individual data and non-standardized reporting of CD4 cell count categories. However, some included studies [21–25] reported significantly more treatment failure (CIN2+/HSIL) in women with lower current and/or nadir CD4 cell counts, which is in agreement with the known increased risk of HPV-related disease according to the severity of immunosuppression [4].

Another limitation of the data available is that only 10 of 40 studies distinguished residual disease from recurrent lesions, and different definitions were used [21–28]. Given this issue, no distinction between residual and recurrent disease was made in this analysis, even if we recognize that these 2 types of disease may have different cancer risk profiles. Better provision might be made for standardized reporting in future studies, for which a pragmatic approach could be to distinguish those lesions detected within the first 12 months from those occurring later. Lastly, a proportion of post-treatment lesions, particularly those of any grade, could be incident lesions, which would be expected to accumulate more frequently in HIV-infected than HIV-uninfected populations [4], but there was no evidence of the significantly higher detection of lesions in studies with longer follow-ups.

In high-resource countries, follow-up after treatment of CIN2+ lesions consists of annual or more frequent visits until 2 consecutive, negative smears [60, 61]. However, this may not be feasible in resource-limited countries, and there is a need to identify and assess simplified follow-up strategies. Data on the performance of VIA for the detection of recurrent lesions is scarce, and additional research should be conducted to determine the value of VIA during follow-up. Of note, although there were 2 studies in which initial lesions were detected using VIA but post-treatment lesions were assessed by histology that met our inclusion criteria (Table 1), we did not include 3 other studies that assessed treatment outcomes by VIA only [15, 18, 62], given VIA’s lack of acceptance as a gold standard of disease ascertainment. In the study by Orang’o et al, the sensitivity of VIA to detect recurrence was as low as 27% [50]. In contrast to our findings, none of these studies found significant differences between HIV-infected and -uninfected participants. HPV persistence is an important risk factor for recurrent cervical disease [63, 64]. In the context of high-resource countries, HPV testing has been proposed as a test of treatment success [65]. On the other hand, in sub-Saharan African women, particularly those infected with HIV, HPV prevalence and incidence rates are very high, such that a follow-up test may have too low a specificity, particularly in the absence of genotyping to identify type-specific persistence [28]. Furthermore, in the limited-resource context of many African countries, cost-effectiveness and the optimal timing of HPV testing need to be determined. Only 2 of the 40 included studies reported on HPV testing post-treatment in HIV-infected women [28, 47], precluding any possibility for a meta-analytical comparison.

In conclusion, this study provides evidence for an increased risk of high cervical lesions post-treatment in HIV-infected women, in comparison to their HIV-uninfected counterparts. Thus, there is a need to reflect upon appropriate follow-up strategies for these women, particularly in limited-resource contexts, where the HIV epidemic is known to be concentrated.

## Supplementary Data

Supplementary materials are available at *Clinical Infectious Diseases* online. Consisting of data provided by the authors to benefit the reader, the posted materials are not copyedited and are the sole responsibility of the authors, so questions or comments should be addressed to the corresponding author.

ciy1123_suppl_Supplementary_Material

## Notes

***Acknowledgments.*** The authors thank Dr Nguyen and Dr Fazzari for their kind help.

***Potential conflicts of interest.*** All authors: No reported conflicts. All authors have submitted the ICMJE Form for Disclosure of Potential Conflicts of Interest. Conflicts that the editors consider relevant to the content of the manuscript have been disclosed.
