# Supplementary material for: Residual or Recurrent Precancerous Lesions After Treatment of Cervical Lesions in Human Immunodeficiency Virus–infected Women: A Systematic Review and Meta-analysis of Treatment Failure
Source: Clin Infect Dis. 2019 Jan 2;69(9):1555–65. doi: 10.1093/cid/ciy1123 (PMC6792085; doi:10.1093/cid/ciy1123)

Residual or recurrent precancerous lesions after treatment of cervical lesions in HIV-infected women: a systematic review and meta-analysis of treatment failure

Pierre Debeaudrap, Joelle Sobngwi, Pierre-Marie Tebeu, Gary M. Clifford

Supplementary Material

Data

- **Supplementary Data S1.** Medline search.

Table

- **Supplementary Table S1.** Characteristics of studies fully reviewed but not included in the meta-analysis, by reason for non-inclusion.

Figures

- **Supplementary Figure S1.** Post-treatment prevalence of cervical lesions in HIV-infected women treated for $\geq$CIN2/HSIL, by sub-group.
- **Supplementary Figure S2.** Funnel plots for meta-analysis of post-treatment lesions in HIV-infected versus uninfected women (left: any grade lesion, right: high-grade lesion).

Supplementary Data S1. Medline search.

(Uterine cervical neoplasms OR cervical intraepithelial neoplasia OR CIN[All Fields] OR ((cervical OR cervix) AND (cancer OR tumor OR tumour OR malignan* OR neoplas* OR carcinoma* OR adenocarcinoma* OR precancer* OR dysplasia))) AND (surgery OR (lletz OR leep) OR (conisation OR conization) OR laser OR excis* OR cryotherapy OR cold coagulation OR cold coagulation OR thermocoagulation OR thermal coagulation OR treatment[MeSH Terms] OR therapeutics[MeSH Terms]) AND (("recurrence"[MeSH Terms] OR recurrence[Text Word]) OR residual[All Fields] OR follow up) AND (HIV Infections[MeSH Terms] OR HIV[MeSH] OR HIV[tiab] OR acquired immune deficiency syndrome virus OR human immunodeficiency virus OR human immunodeficiency viruses OR aids).

Supplementary Table S1. Characteristics of studies fully reviewed but not included in the meta-analysis, by reason for non-inclusion.

Study author, publication year	Setting & period	Treatment modality	Type of lesion treated[a]	Post-treatment outcome criteria	Number of subjects	
					HIV+	HIV-
Follow-up (maximum or median) <6 months						
Adam, 2008	South Africa (2003 – 2006)	LEEP	High-grade	Cyto	575	-
Batra, 2010	South Africa (2006 – 2009)	LEEP	Any grade	Cyto	219	275
Malapati, 2011	US (2004 – 2008)	LEEP	Any grade	Cyto & histo	118[b]	
Mutyaba, 2010	Uganda (2007 – 2008)	Mixed/other	Any grade	VIA & histo	464[b]	
Unavailability of relevant data (numbers treated and/or post-treatment outcomes) on HIV-infected women						
Serati, 2012	Italy (1999 – 2009)	Mixed/other	Any grade	Cyto & histo	4	278
English, 2014	US (1999 – 2011)	Mixed/other	High-grade	Cyto	118[b]	
Malapati, 2011	US (2004 – 2008)	LEEP	Any grade	Cyto & histo	464[b]	
Mutyaba, 2010	Uganda (2007 – 2008)	Mixed/other	Any grade	VIA & histo	29[b]	
Bambury, 2013	Jamaica (1994 – 2004)	Mixed/other	Any grade	Cyto	15	21

Belmonte, 2003	Spain[c]	LEEP	Any grade	Histo	71	80
Frega, 2006	Italy (1997 – 1999)	LEEP	High-grade	Cyto & histo	41	45
Ferrero, 2002	Italy (1991 – 2001)	NA	NA	Cyto & histo	23	-
Isaakidis, 2013	India (2010)	Mixed/other	Any grade	Cyto & histo	95	-
Joshi, 2013	India (2010 – 2011)	Mixed/other	Any grade	VIA & histo	53	-
<u>Absence of cytological and/or histological ascertainment of post-treatment outcome</u>						
Campbell, 2016	Malawi (2013 – 2015)	Thermoablation	VIA-positive	VIA	11	26
Martin, 2014	Guyana (2009 – 2012)	Mixed/other	VIA-positive	VIA	123	904
Oga, 2016	Nigeria (2010 – 2014)	Thermoablation	VIA-positive	VIA	120	57
<u>Populations selected upon post-treatment outcome</u>						
De Mello Silva, 2014	Brazil (2004 – 2011)	LEEP	High-grade	Histo	5[#]	95[#]
Van Bogaert, 2015	South Africa (2008 – 2013)	LEEP	High-grade	Histo	72[§]	104[§]

Abbreviations: Cyto & histo, cytology and histology; Mixed/other, use of different treatments among cryotherapy, thermal ablation or loop electrosurgical excision procedure (LEEP) and/or of other treatment; VIA & histo, visual inspection with acetic acid and histology.
[a]High-grade lesion: cervical intraepithelial neoplasia 2+ (CIN2+) or high-grade squamous intraepithelial lesion (HSIL) lesion;[b]Number of participants infected and uninfected with HIV not available; [c]Year of the study not available.
[#]case control design: 50 with treatment failure versus 50 without treatment failure. [§]Women with involved margins post-LEEP only.

References to Supplementary Table S1

Adam Y, van Gelderen CJ, de Bruyn G, McIntyre JA, Turton DA, Martinson NA. Predictors of persistent cytologic abnormalities after treatment of cervical intraepithelial neoplasia in Soweto, South Africa: a cohort study in a HIV high prevalence population. BMC cancer **2008**; 8: 211.

Bambury I, Mullings A, Fletcher H, Johnson N, Tolluch-Reid M. Cervical intraepithelial neoplasia in a cohort of HIV-positive women at the University Hospital of the West Indies: management and outcome. West Indian Med J **2013**; 62(4): 313-7.

Batra P, Kuhn L, Denny L. Utilisation and outcomes of cervical cancer prevention services among HIV-infected women in Cape Town. S Afr Med J **2010**; 100(1): 39-44.

Belmonte S, Garcia A, Hermandez J, et al. Recurrencia de la neoplasia cervical intraepithelial en pacientes VIH positivas. Toko Gin Pract **2003**; 62(4): 274 - 8.

Campbell C, Kafwafwa S, Brown H, et al. Use of thermo-coagulation as an alternative treatment modality in a 'screen-and-treat' programme of cervical screening in rural Malawi. International journal of cancer Journal international du cancer **2016**; 139(4): 908-15.

de Mello Silva MV, Coutinho IC, de Andrade Heraclio S, Fittipaldi HM, Jr., Katz L. Factors associated with the persistence/recurrence of CIN2/3 in women submitted to loop electrosurgical excision procedure in a teaching hospital in northeastern Brazil: a case-control study. Journal of lower genital tract disease **2014**; 18(4): 286-90.

English DP-g, Pasternak M, Warmington J, et al. Evaluation of the diagnostic accuracy of cervical biopsy and determination of associated risk factors for positive margin status in recurrent cervical dysplasia after leep or conization. Journal of Clinical Oncology **2014**; 32(15_suppl): 5609-.

Ferrero S, Arena E, De Masi E, Biasotti B, Fulcheri E, Bentivoglio G. Screening and treatment for cervical intraepithelial neoplasia (CIN) in HIV-infected women. Minerva ginecologica **2002**; 54(4): 297-307.

Frega A, Biamonti A, Maranghi L, et al. Follow-up of high-grade squamous intra-epithelial lesions (H-SIls) in human immunodeficiency virus (HIV)-positive and human papillomavirus (HPV)-positive women. analysis of risk factors. Anticancer Res **2006**; 26(4b): 3167-70.

Isaakidis P, Pimple S, Varghese B, et al. HPV infection, cervical abnormalities, and cancer in HIV-infected women in Mumbai, India: 12-month follow-up. Int J Womens Health **2013**; 5: 487-94.

Joshi S, Sankaranarayanan R, Muwonge R, Kulkarni V, Somanathan T, Divate U. Screening of cervical neoplasia in HIV-infected women in India. AIDS (London, England) **2013**; 27(4): 607-15.

Malapati R, Chaparala S, Cejtin HE. Factors influencing persistence or recurrence of cervical intraepithelial neoplasia after loop electrosurgical excision procedure. J Low Genit Tract Dis **2011**; 15(3): 177-9.

Mutyaba T, Mirembe F, Sandin S, Weiderpass E. Evaluation of 'see-see and treat' strategy and role of HIV on cervical cancer prevention in Uganda. Reprod Health **2010**; 7: 4.

Martin CE, Tergas AI, Wysong M, Reinsel M, Estep D, Varallo J. Evaluation of a single-visit approach to cervical cancer screening and treatment in Guyana: feasibility, effectiveness and lessons learned. J Obstet Gynaecol Res **2014**; 40(6): 1707-16.

Oga EA, Brown JP, Brown C, et al. Recurrence of cervical intraepithelial lesions after thermo-coagulation in HIV-positive and HIV-negative Nigerian women. BMC women's health **2016**; 16: 25.

Serati M, Siesto G, Carollo S, et al. Risk factors for cervical intraepithelial neoplasia recurrence after conization: a 10-year study. European journal of obstetrics, gynecology, and reproductive biology **2012**; 165(1): 86-90.

van Bogaert LJ. Involved LEEP excision margins as predictor of residual/recurrent disease in HIV-positive and HIV-negative women in a low-resource setting. Anal Quant Cytopathol Histpathol **2015**; 37(2): 105-8.

Supplementary Figure S1. Post-treatment prevalence of cervical lesions in HIV-infected women treated for >CIN2/HSIL, by sub-group.

	High-grade			Any grade		
	Studies	Subjects	Event	Studies	Subjects	Event
**Treatment method**						
LEEP[a]	6	594	80	4	234	119
Cryotherapy[a]	3	195	44	4	223	121
Mixed/other[a]	9	756	263	13	871	535
**Follow−up duration**						
>12 months[b]	9	494	158	12	545	318
6 to 12 months[b]	6	990	215	6	722	427
**Income setting**						
High−income	9	495	166	14	622	357
Low−income	7	1050	221	5	706	418
**HIV−uninfected controls**						
With	6	453	100	9	512	253
Without	10	1092	287	10	816	522
**Year of publication**						
≤ 2005	4	212	35	8	280	115
>2005	12	1333	352	11	1048	660
**Overall**						

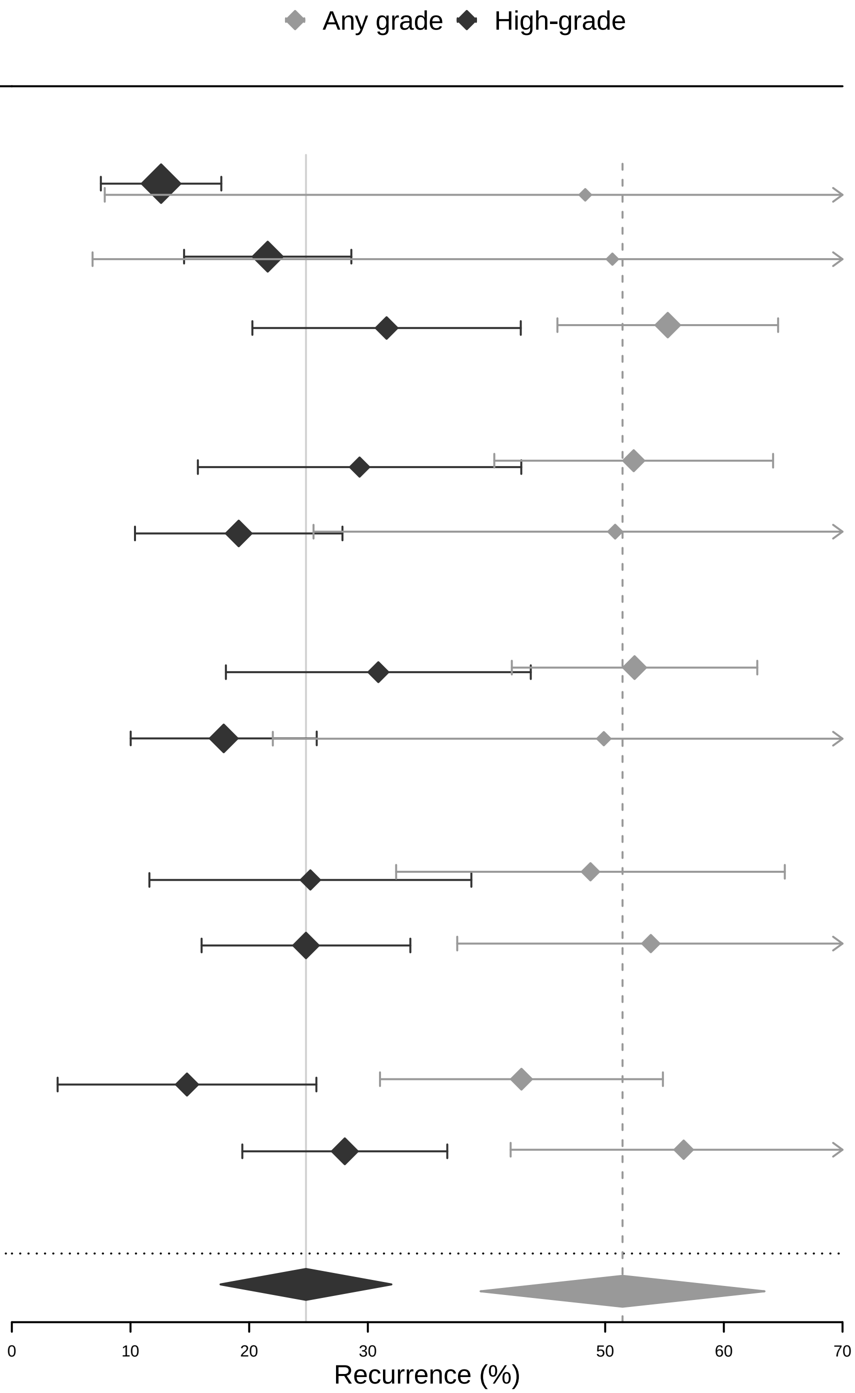

[a]Number of studies do not add up to the total because one study may contribute to multiple categories. [b]One study has missing information.

Abbreviations: LEEP, loop electrosurgical excision procedure; Mixed/other, use of different treatments among cryotherapy, thermal ablation or LEEP and/or of other treatment.

Supplementary Figure S2. Funnel plots for meta-analysis of post-treatment lesions in HIV-infected versus uninfected women (left: any grade lesion, right: high-grade lesion).

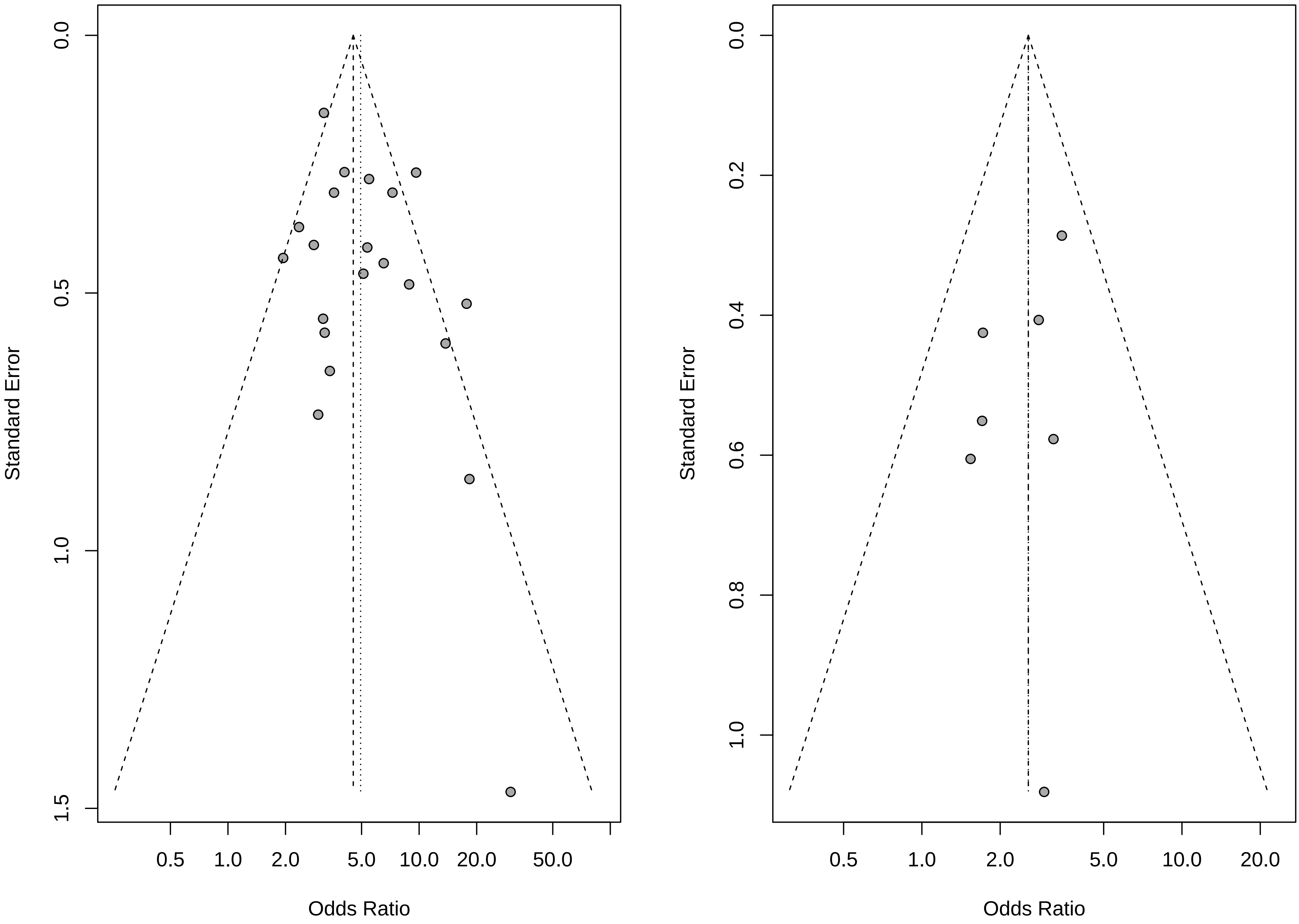

Supplement: ciy1123_suppl_Supplementary_Material [file ciy1123_suppl_supplementary_material.pdf]
